# Vitreoretinal Lymphoma

**DOI:** 10.3390/cancers13163921

**Published:** 2021-08-04

**Authors:** Bianka Sobolewska, Soon-Phaik Chee, Fatma Zaguia, Debra Anne Goldstein, Justine R. Smith, Falko Fend, Manabu Mochizuki, Manfred Zierhut

**Affiliations:** 1 Center of Ophthalmology, University of Tuebingen, 72076 Tuebingen, Germany; Manfred.zierhut@med.uni-tuebingen.de; 2 Singapore National Eye Centre, 11 Third Hospital Avenue, Singapore 168751, Singapore; chee.soon.phaik@singhealth.com.sg; 3 Singapore Eye Research Institute, 11 Third Hospital Avenue, Singapore 168751, Singapore; 4 Department of Ophthalmology, Yong Loo Lin School of Medicine, National University of Singapore, Singapore 168751, Singapore; 5 Duke-NUS Medical School, Singapore 168751, Singapore; 6 Department of Ophthalmology, Feinberg School of Medicine, Northwestern University, Chicago, IL 60611, USA; fatma.zaguia@mail.mcgill.ca (F.Z.); debra.goldstein@northwestern.edu (D.A.G.); 7 College of Medicine & Public Health, Flinders University, Adelaide 5042, Australia; justine.smith@flinders.edu.au; 8 Institute for Pathology and Neuropathology, University of Tuebingen, 72076 Tuebingen, Germany; Falko.Fend@med.uni-tuebingen.de; 9 Miyata Eye Hospital, Miyakonojo, Miyazaki 885-0051, Japan; m.manabu.oph@tmd.ac.jp

**Keywords:** vitreoretinal lymphoma, CNS lymphoma, IL-10/IL-6 ratio, MYD88, methotrexate

## Abstract

**Simple Summary:**

Vitreoretinal lymphoma is a variant of primary CNS lymphoma involving the retina and/or the vitreous. At the time of presentation, CNS involvement occurs in up to one-third of patients. However, 50–90% of patients develop a CNS and/or spinal cord disease within one year. Therefore, it is important to frequently examine and recognize the early symptoms of CNS involvement. This review summarizes the clinical signs, ocular diagnosis and treatment of vitreoretinal lymphoma.

**Abstract:**

Vitreoretinal lymphoma (VRL) is a rare variant of primary central nervous system lymphoma (PCNSL), mostly of diffuse large B cell lymphoma, which affects the retina and/or the vitreous with or without optic nerve involvement. The disease course is aggressive. Up to 90% of the patients develop central nervous system lymphoma within one year. The diagnosis of VRL is challenging due to nonspecific chronic and relapsing uveitis and is made by anterior chamber tab or vitreous aspirate biopsy. There is no established treatment protocol for VRL patients with bilateral involvement without CNS involvement. There are suggestions to use only intravitreal chemotherapy with methotrexate and/or rituximab. Alternatively, systemic high-dose MTX treatment or external beam radiotherapy is used. Further studies are needed to prove and confirm the prophylactic systemic therapy in preventing CNS involvement in limited VRL.

## 1. Introduction and Nomenclature

Intraocular lymphomas may be classified into the following subtypes: vitreoretinal lymphomas (VRL) (the most common subtype) that may be subdivided into primary or secondary VRL; primary uveal/choroidal lymphomas; other secondary intraocular lymphomas. Only a small percentage of VRL are of T cell or natural killer (NK) cell origin. As not all lymphomas with vitreoretinal involvement represent VRL in the strict sense, systemic evaluation is highly important for accurate diagnosis [1].

Primary uveal or choroidal lymphomas are usually low-grade extranodal marginal zone lymphoma (EMZL) of the mucosa-associated lymphoid tissue (MALT) type with excellent prognosis and good response to local radiotherapy. Another treatment option is systemic chemotherapy, e.g., with rituximab [2]. Secondary intraocular lymphomas mainly originate due to hematogenous dissemination from systemic lymphoma. They disseminate predominantly to the uvea which has rich blood supply or, less commonly, to the vitreous or the retina (systemic metastatic retinal lymphoma, SMRL) from a known source of systemic lymphoma, the latter particularly from the nasopharynx, the testes or the skin [1,2,3,4]. Intravascular diffuse large B cell lymphoma (DLBCL) is a rare form of systemic extranodal non-Hodgkin lymphoma characterized by proliferation and aggregation of large malignant B cells within the lumen of small blood vessels, commonly affecting the CNS and also the choroid [5].

### The Relationship of Vitreoretinal Lymphoma and Primary Central Nervous System Lymphoma

VRL may involve the vitreous, the retina, the subretinal space and the optic nerve head. Primary vitreoretinal lymphoma (PVRL) is defined as malignant lymphoproliferation solely involving the retina, the vitreous or both structures. PVRL is regarded as a variant of primary CNS lymphoma (PCNSL) based on frequent synchronous or metachronous manifestations at both sites and similar clinical behavior with rare dissemination outside of the CNS as described below [6,7]. The majority of primary central nervous system lymphomas (PCNSL) and 95% of VRL are classified as diffuse large B cell lymphoma (DLBCL), which also is the most common non-Hodgkin lymphoma overall [7,8,9,10,11,12,13]. Secondary VRL mostly occurs in the setting of PCNSL and is less frequent following systemic DLBCL, of which testicular lymphoma is the most common [13,14].

Recent molecular studies have elucidated the genetics, immunogenetics and cell of origin of VRL, but much of the information is derived from analogy with PCNSL, and only a handful of studies have investigated VRL proper [15,16,17,18,19,20,21,22,23,24,25,26,27]. VRL, like PCNSL, mostly belongs to the activated B cell (ABC) type of DLBCL based on immunophenotype, gene expression and mutational profile. Until recently, little was known about the genetic profile of VRL, but a high frequency of *MYD88* and *CD79B* mutations has been observed and exploited for diagnostic purposes as described in more detail in the diagnostic section [15,18,25]. Recently, two large-scale genomic studies of systemic DLBCL have identified a specific subgroup of ABC with common extranodal manifestations, poor prognosis, activation of immune evasion mechanisms and high frequency of mutations caused by aberrant somatic hypermutation [28,29]. This subgroup, called *MYD88/CD79B*-mutated (MCD) or cluster 5, is dominant at so-called immune-privileged sites including the testes and the CNS [30,31]. Recently, it has been shown that both primary and secondary VRL, similarly to PCNSL, belong to the MCD/cluster 5 subgroup of DLBCL, with high frequencies of mutations in *MYD88*, *CD79B*, *PIM-1*, *IGLL5*, *BTG1/2*, *TBL1XR1* and *ETV6*, as well as common deletions of 9p21/*CDKN2A*. Of note, secondary VRL following systemic DLBCL shows an identical genetic profile, indicating that secondary spread to the vitreoretinal space is a result of the biological properties of this DLBCL subtype rather than a chance occurrence.

As mature B cell lymphomas, VRL carry highly somatically mutated IGH genes compatible with the post-germinal center origin/ABC type of DLBCL [21]. A recent study has demonstrated a very restricted IG gene repertoire, with high frequency of IGHV4-34 gene usage and a subset of cases with stereotyped IGHV3-7 rearrangements [32].

Given the similarities between primary VRL and PCNSL and thus secondary VRL, in this review, we therefore avoid using the term “primary VRL.” However, the eye can be the first manifestation of this lymphoma which may lead to different treatment strategies, therefore we use that term when we refer to different therapy strategies later in this chapter (“treatment chapter”). The term “primary intraocular lymphoma” (PIOL) has been replaced by “VRL” since 2009 due to the distinctive features of lymphomas which originate from the retina/vitreous versus the choroid (see below) [8,33].

## 2. Epidemiology and Pathogenesis

The incidence of PCNSL and VRL is on the increase globally [34,35,36]. In the past three decades, the incidence of PCNSL appears to have tripled as seen in the Finnish Cancer Registry and in the USA [37]. Levasseur et al. [38] reported that the incidence of VRL in British Columbia doubled from 1990 to 2010, achieving 0.047 cases per 100,000 people per year [38]. In the US, it is estimated that there are approximately 380 new cases of VRL per year [39]. The real incidence is still undetermined due to lack of a central registry [35].

While PCNSL accounts for 4–6% of all brain tumors and less than 1% of all non-Hodgkin lymphomas [40,41], VRL is rare, accounting for less than 0.01% of all ocular diagnoses [41] and 1.1% of uveitis cases at a referral clinic [42].

At the time of presentation with VRL, CNS involvement is present in 16–34% of patients [39,43]. On the other hand, 15–20% of all PCNSL patients have VRL at presentation; 50–90% of patients with VRL develop a CNS and/or spinal cord disease after 16–24 months [40,44,45]. In a large retrospective study, the median interval of onset between ocular and CNS DLBCL was 21.7 months (range: from 1 week to 10 years) [46]. These data highlight the close relationship between VRL and PCNSL and indicate that improved diagnostic methods at regular intervals result in an increased detection rate of CNS involvement in patients with primary VRL. In immunosuppressed individuals, especially in patients with the HIV infection, PCNSL and, by extension, VRL are almost invariably associated with a latent infection of B cells by the Epstein–Barr virus [47,48,49]. In addition, the incidence of PCNSL is significantly higher in patients with AIDS than in immunocompetent patients [50].

VRL typically occurs in older individuals between 50 and 70 years of age, with the median age of 60 years [8,51], ranging from 36–90 years [52]. In the immunosuppressed, it is not unusual for VRL to present at a younger age. Some authors have reported a slight male gender predilection, with the male/female ratio of 1.2–1.7:1 [53], while more recent publications found a slight female predominance [7,40,52,54].

## 3. Clinical Features

Recently, “Consensus Recommendations for the Diagnosis of VRL” have been published, which were established using a Delphi procedure (Table 1) [55].

**Figure 1:**
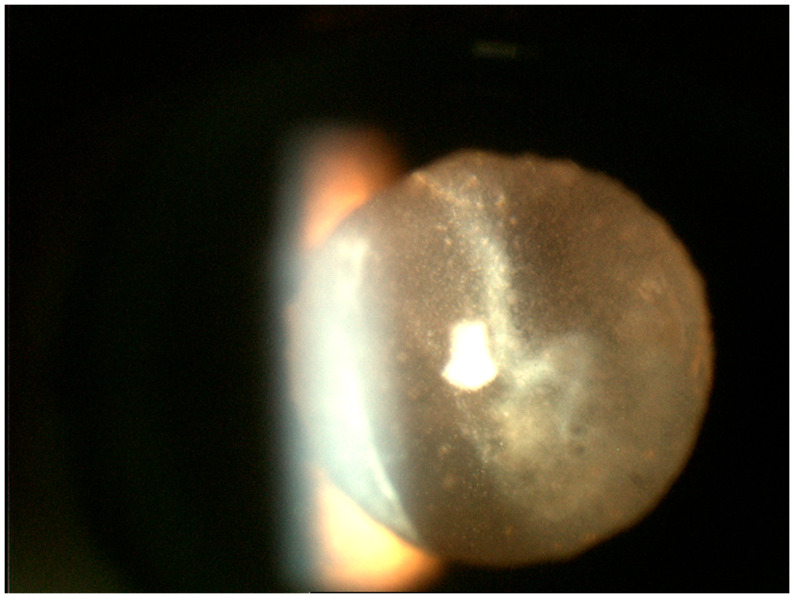
Slit lamp photograph of the retrolental region demonstrating sheets of numerous white cells of varied sizes infiltrating the vitreous. Most of these lymphoma cells tend to be larger than what is seen in vitritis.

**Figure 2:**
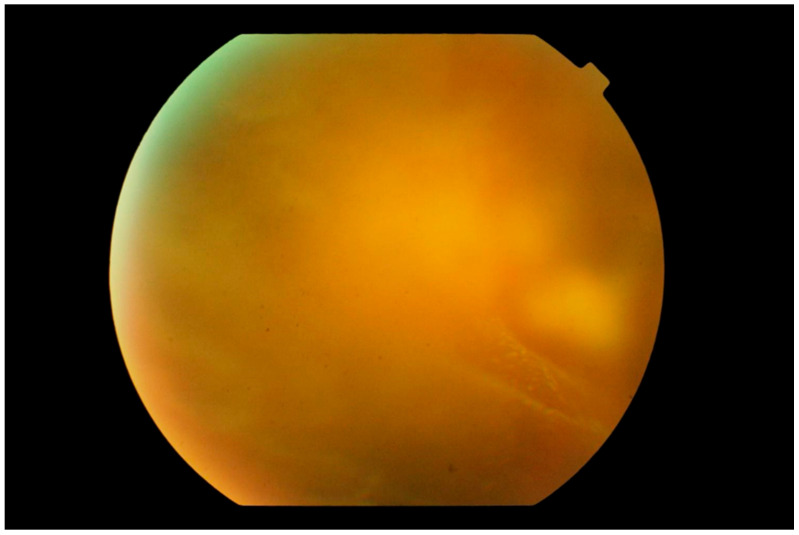
Photograph of the peripheral fundus showing the “aurora borealis” sign caused by lymphoma cell infiltration along the vitreous fibrils creating streaks of opacity. In the background, a multinodular yellowish mass is just visible.

More than 80% of VRL patients have bilateral involvement [56]. The disease typically presents insidiously as nonspecific chronic and relapsing uveitis with floaters, painless moderate blurring of vision and minimal ocular discomfort [46,57]. Clinical examination reveals numerous vitreous cell sheets which are the most common finding and the hallmark feature of VRL [58] (Figure 1). These cells tend to be larger in size but less in density that those seen in vitritis. A study of the vitreous in 26 eyes of 13 patients with VRL found that vitreous haze was present in 92% of the eyes and was the only sign of VRL in 15%. Three nonexclusive patterns of vitreous haze identified included an “aurora borealis” pattern (46%), a nonspecific pattern (38%) and a string-of-pearls pattern (23%). The pattern manifested may be related to the state of the vitreous. The aurora borealis pattern (Figure 2) is observed on indirect ophthalmoscopy or on slit lamp examination as a unique arrangement of vitreous haze resulting from large cells being aligned along a radial wave-like fibril pattern in the peripheral vitreous, especially in the superior peripheral vitreous [39]. This may be observed in eyes with a relatively preserved vitreous structure. The nonspecific vitreous haze is more likely seen in eyes with more liquefied vitreous humor. The least common pattern is where the tumor cells cling onto fine fibrils of the vitreous, forming a string-of-pearls appearance. These pearls, which are not specific for diagnosis, may also be seen following vitrectomy in VRL eyes as well as in sarcoidosis and infectious uveitis such as toxoplasmosis [59].

**Figure 3:**
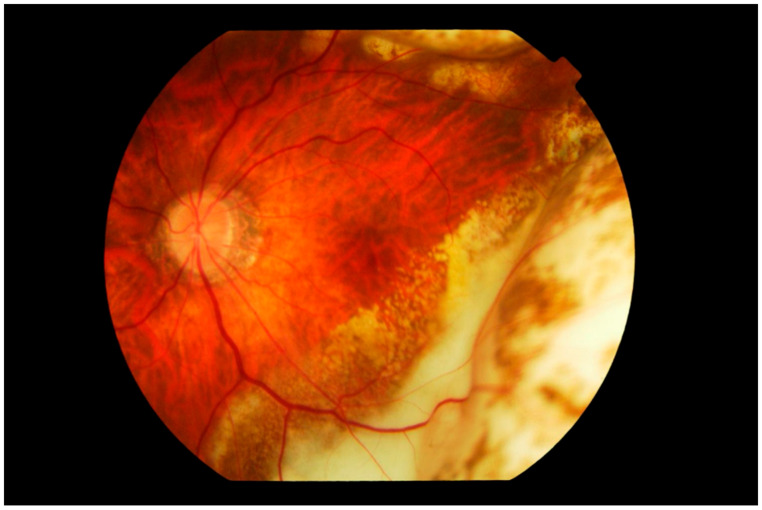
Fundus photograph of the left eye showing subretinal infiltration by lymphoma cells forming multiple large yellowish cream nodular masses distributed circumferentially with patchy pigmentation, giving a characteristic “leopard spot” appearance. The inferior temporal fundus shows the recent development of a wide span of subretinal infiltrates, contiguous with the longer standing peripheral mass. The edge of the lesion advancing towards the fovea consists of multiple small cream-colored spots. The vitreous is clear.

**Figure 4:**
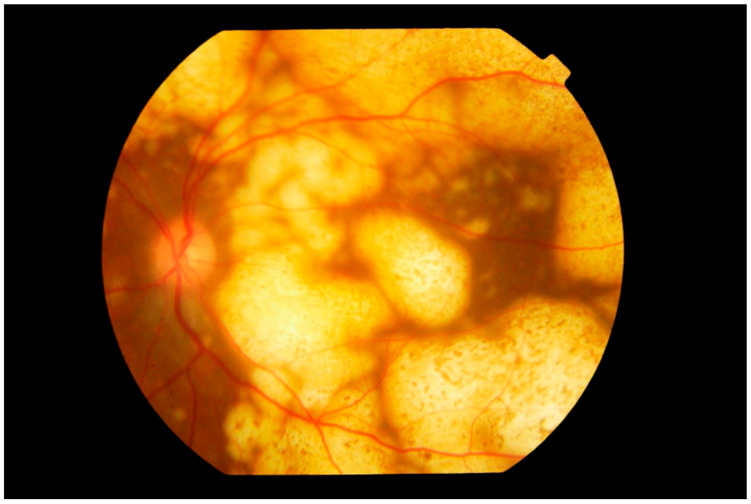
Fundus photograph of the left eye showing extensive involvement of the entire fundus by multiple nodular subretinal lymphoma masses of varying sizes. The larger peripheral lesions are formed by the merging of small lesions and have pigmentary change, giving a “leopard spot” appearance. The multiple yellow creamy lesions superior temporal to the disc which are smaller and more recent are also subretinal in location.

**Figure 5:**
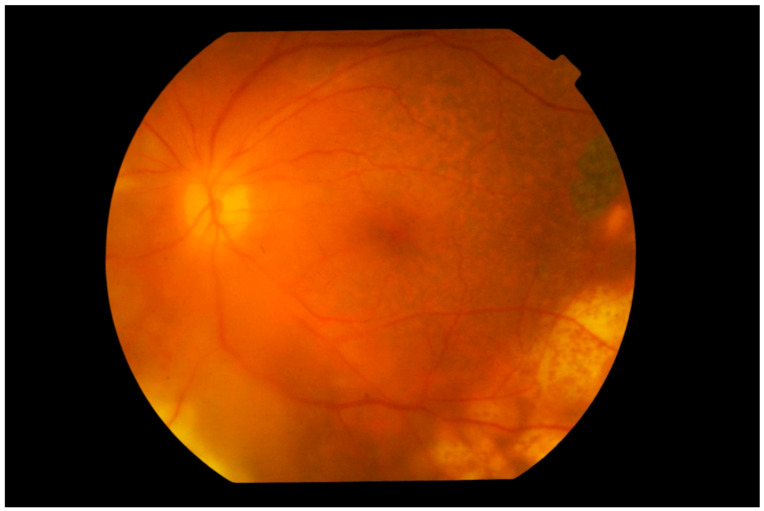
Photograph of the left fundus showing multiple yellowish nodular subretinal lesions with “leopard spot” pigmentation inferior temporally. Temporally to the fovea and in the superior temporal fundus, pigmentary mottling of the retina representing previous spontaneously resolved vitreoretinal lymphoma lesions is clearly seen. The vitreous is hazy but the disc appears normal.

The presence of multifocal yellowish-white/cream-colored subretinal [60] or retinal lesions is a characteristic feature of VRL (Figure 3). These lesions may be found as a cluster anywhere in the fundus and may increase in size over time or spontaneously resolve, leaving retinal pigment epithelium (RPE) atrophy and subretinal fibrosis [61,62,63] (Figure 4). These features are observed in 50% of cases at presentation [46,57] and may take on a “leopard spot” appearance (Figure 5), formed by the collection of subretinal pigmented lesions that coalesce over time [39].

**Figure 6:**
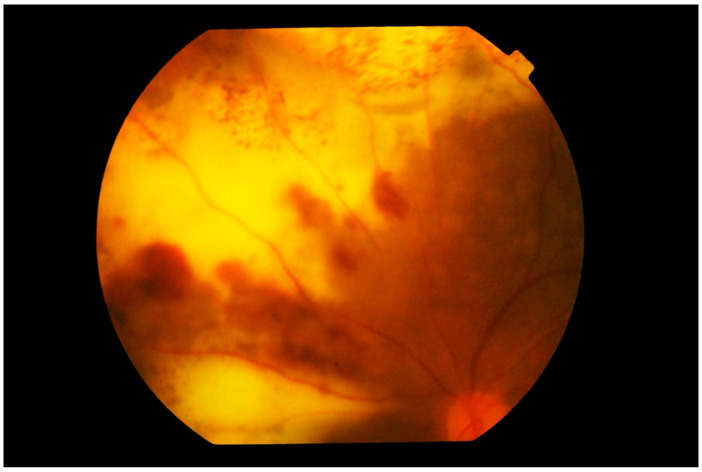
Photograph of the left superior nasal fundus showing large confluent yellowish subretinal infiltration by lymphoma cells. Large blotches of retinal hemorrhage are also observed, creating a picture of deep retinal necrosis. Note that the vitreous is clear and the disc is not swollen.

Other less common findings include necrotizing retinal infiltrates, large areas of yellow creamy infiltrates, retinal vasculitis, retinal hemorrhages (Figure 6), retinal artery occlusion and optic nerve infiltration [46,48,59,64,65]. Serous retinal detachments may also rarely be present [66,67,68].

Uncommonly, VRL may present with multiple small punctate whitish lesions distributed over the fundus at the outer retina/RPE level, and may be mistaken for multiple evanescent white dot syndrome [69,70]. Typically, minimal or no macular edema is present, accounting for the better than expected visual acuity in spite of significant vitreous haze. In one study of 22 patients with VRL, 50.0% presented with vitreous opacity without retinal lesions, 36.4% presented with sub-RPE infiltration, 9.1%—with multiple small whitish spots, 4.6%—with necrotizing and hemorrhagic retinitis [69].

**Figure 7:**
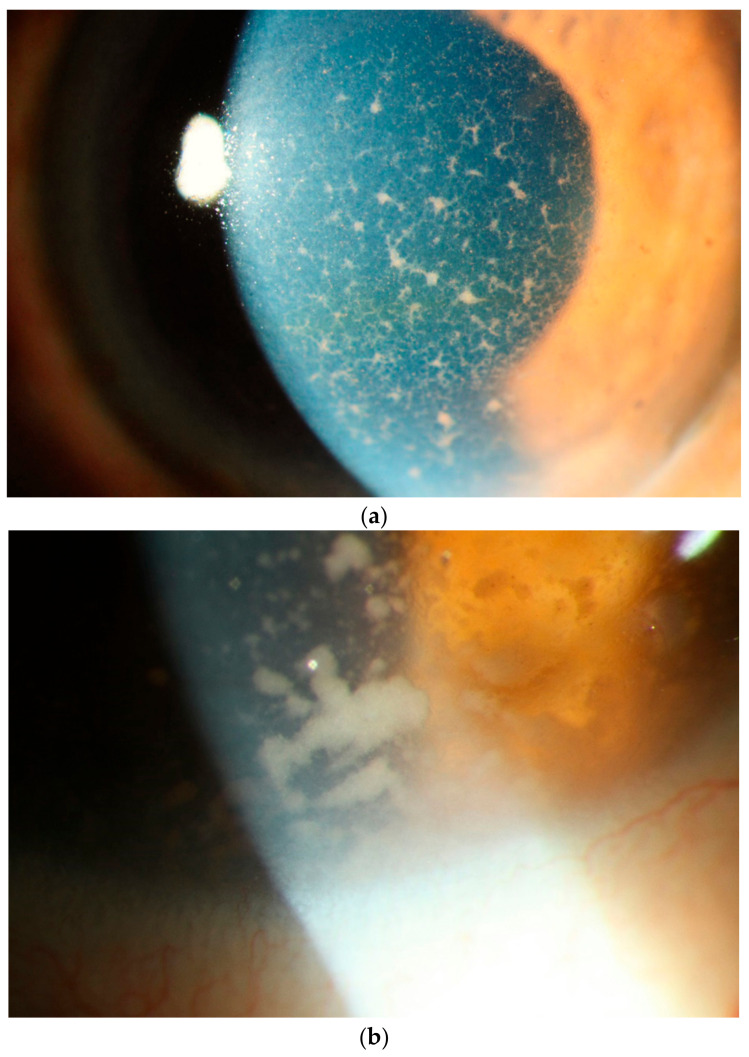
Slit lamp photograph showing (**a**) diffusely distributed keratic precipitates (KP) on the corneal endothelium. These KPs are a mixture of small and fine or infiltrative KP intermixed with some granulomatous KP. Many of these granulomatous KP have fibrillar extensions, taking on a comet-like appearance, typical of vitreoretinal lymphoma. These faintly pigmented KP may be mistaken for the KP of viral anterior uveitis. (**b**) Occasionally one can find even larger tumor cell collections on the endothelium.

In addition to posterior segment manifestations, there may be nongranulomatous keratic precipitates (which may be dendritiform and diffusely distributed) [71], anterior chamber cells and minimal flare in the anterior segment [72,73] (Figure 7a). Occasionally patients may present with large clumps of malignant cells on the corneal endothelium, mimicking granulomatous KP (Figure 7b). Corneal edema, pseudohypopyon [54,74] and pupil distortion may be present but are not common [65]. As the anterior segment inflammation is minimal, photophobia, pain and redness are uncommon symptoms [6].

Thus, VRL often masquerades as a nonspecific chronic intermediate or posterior uveitis [75,76]. It responds initially to oral corticosteroids, but later becomes steroid-dependent or refractory to immunosuppressive treatment [8,58]. This partial response to corticosteroids may lead to further delay in diagnosis. On average, it takes over 12 months between the onset of symptoms and clinching the diagnosis of VRL [39,77]. Early local treatment of VRL is unlikely to influence eventual CNS involvement, However, systemic treatment of the eye before CNS involvement has been shown to prolong survival [78]. This delay in treatment often results in some patients going on to develop a CNS disease, worsening the overall prognosis. The most useful signs for differentiating VRL from other causes of uveitis include better than expected visual acuity, minimal anterior chamber flare, absence of posterior synechiae, homogeneity of the vitreous body without inflammatory stranding or destruction of the vitreous architecture, absence of associated cystoid macular edema, lack of disc swelling and epiretinal membrane [57,79]. Furthermore, the presence of migrating retinal lesions, especially in the absence of vitritis, and spontaneous resolution of large subretinal lesions should raise the index of suspicion for VRL [80]. Spontaneous tumor resolution is hypothesized to be a result of host tumor control by CD8+ T cells and natural killer cells [81].

Ocular involvement may precede, be concomitant to or follow a CNS disease. As the majority of VRL patients develop a CNS disease, it is important to frequently screen for it, as well as recognize the early symptoms. On average, there is a 3-month delay between the initial CNS symptoms and the diagnosis of CNS lymphoma [77]. CNS involvement may present with personality change, cognitive decline and manifestations due to intracranial hypertension such as headache, nausea and vomiting [82]. Similar to VRL, PCNSL initially responds to corticosteroids, which may lead to misdiagnosis and delay in appropriate treatment.

### Differential Diagnosis

VRL presenting as chronic relapsing intermediate or posterior uveitis with nonspecific symptoms and signs may be misdiagnosed as uveitis syndrome or idiopathic uveitis. These include such infections as chronic endophthalmitis, acute retinal necrosis, cytomegalovirus retinitis, retinochoroidal toxoplasmosis, syphilitic retinitis, pneumocystis choroiditis, coccidioidal choroiditis, noninfectious uveitis such as birdshot choroidopathy, serpiginous choroiditis, Vogt–Koyanagi–Harada syndrome, acute posterior multifocal placoid pigment epitheliopathy, sarcoidosis, sympathetic ophthalmitis, Whipple’s disease or other infectious or malignant uveitis [65,83,84,85]. Neoplastic conditions include amelanotic melanoma, choroidal lymphoma, metastatic malignancy and uveal lymphoma.

## 4. Diagnostics

### 4.1. Imaging

The diagnosis of VRL can be challenging and is often delayed, which may result in increased mortality and morbidity. Noninvasive imaging modalities can contribute to an earlier detection of the disease. In the following section, we present characteristic findings of VRL with fundus autofluorescence (FAF), spectral domain optical coherence tomography (SD-OCT), fluorescein angiography (FA) and indocyanine green angiography (ICGA).

#### 4.1.1. Fundus Autofluorescence

**Figure 8:**
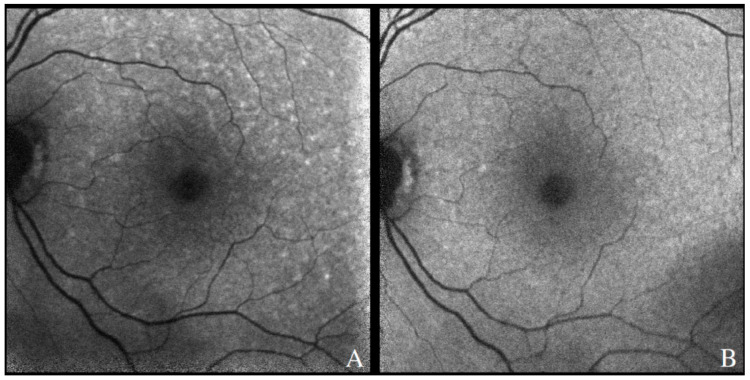
Fundus autofluorescence demonstrating hyperautofluorescent spots in the posterior pole of a patient with recurrence of PVRL (**A**). There was resolution after therapy involving systemic chemotherapy, with few residual hypoautofluorescent spots along the superior arcade (**B**).

The features seen with FAF in patients with VRL can vary. In the majority of eyes, a pattern of granular hyper- and hypoautofluorescence can be seen, particularly in those with active disease [86]. Hyperautofluorescent spots likely represent areas of active disease, with lymphomatous infiltration and secondary RPE dysfunction, while hypoautofluorescent spots suggest VRL cells above the RPE or RPE atrophy [39]. Hyperautofluorescent lesions have been described to correlate with hypofluorescent spots in FAF images and hyperreflective sub-RPE infiltrates in OCT scans [86,87]. More importantly, hyperautofluorescence in FAF images has been shown to regress upon treatment of disease [88], suggesting that FAF can be helpful in following the response to treatment or detecting recurrences in a patient with a known VRL diagnosis (Figure 8).

#### 4.1.2. Spectral Domain Optical Coherence Tomography 

**Figure 9:**
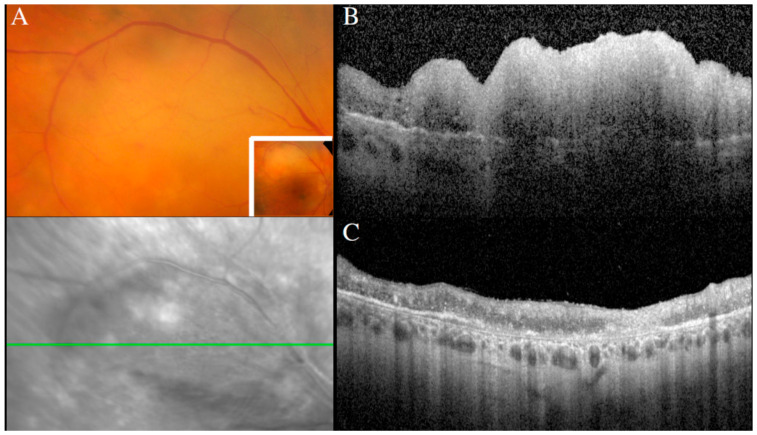
Creamy retinal lesion on a fundus photograph (**A**), corresponding to a large inner retinal infiltrate on a SD-OCT scan (**B**). Regression of the retinal lesion after intravitreal methotrexate, with residual retinal atrophy (**C**).

**Figure 10:**
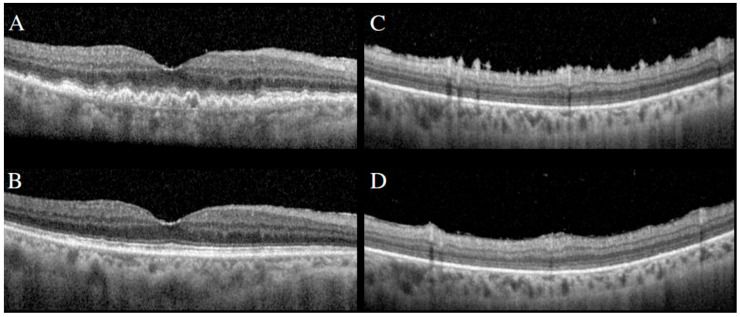
SD-OCT demonstrating large sub-RPE infiltrates (**A**) and preretinal/inner retinal spike-like lesions (**C**). Following intravitreal methotrexate, resolution of sub-RPE infiltrates (**B**) and spike-like lesions (**D**).

There is a wide range of features in OCT that can be observed in VRL, all of which can be present in an individual patient at a single timepoint [89] or at different stages of disease [88]. Most commonly, OCT findings include inner and outer retinal infiltrates [89,90] (Figure 9), sub-RPE infiltration between the RPE and the Bruch’s membrane [89,90] (Figure 10A,B), loss of outer retinal laminations/disruption of the ellipsoid layer, inner retinal spike-like lesions [5] (Figure 10C,D) and vertical hyperreflective lesions [91]. In cases where the disease clinically appears unilateral, hyperreflective foci in the vitreous gel of the contralateral (otherwise quiescent) eye can also serve as clue for a bilateral process.

SD-OCT can also aid in differentiating VRL from other infiltrative malignant processes such as choroidal lymphoma, which may be difficult to do with clinical examination alone. Infiltration in VRL typically occurs between the RPE and the Bruch’s membrane [57,89]; in contrast, choroidal lymphoma typically occurs deep to the RPE–Bruch’s complex [92].

#### 4.1.3. Fluorescein Angiography

Several patterns have been described in FA images in patients with VRL. One of the most common patterns seen is hypofluorescent round spots with the classic “leopard spot” appearance [56,85]. Lesions appear as clusters of small round hypofluorescent spots, measuring between 50 to 250 μm in diameter. These hypofluorescent lesions correspond to deep white retinal lesions seen in fundus photographs and remain hypofluorescent in the late phases of FA [57]. This pattern is likely due to primary retinal lymphoma infiltrates between the Bruch’s membrane and the RPE and can be seen in both the posterior pole and in the retinal periphery. Hallmarks of uveitis such as cystoid macular edema (CME) associated with petaloid macular leakage and posterior pole retinal vasculitis are rarely seen in patients with VRL [57,93].

More recent publications have addressed the role of ultra-widefield imaging in further characterizing the angiographic findings of VRL [93]. Peripheral retinal vasculitis has been seen in up to 77% of cases, which is significantly more than previously reported (6–36%) [52,57,93]. The higher prevalence is likely due to enhanced detection compared with standard 30° and 55° fluorescein imaging. Another pattern noted in ultra-widefield FA images is peripheral small vessel leakage localized around small subretinal infiltrates, which can be useful in identifying small peripheral lesions that may be difficult to see clinically. Furthermore, concurrent OCT findings may be helpful in predicting FA patterns. Sub-RPE lesions with no associated intraretinal or subretinal fluid nor retinal disorganization in OCT scans were typically hypofluorescent in FA images. In contrast, lesions with associated overlying retinal disorganization or fluid would typically have associated vascular leakage and appear hyperfluorescent on FA [87,94].

#### 4.1.4. Indocyanine Green Angiography 

The yield of ICGA in early detection and diagnosis of VRL is much lower than of other imaging modalities due to the fact that malignant cells tend to locate to the retina and not the choroid. Small hypofluorescent lesions in ICGA images have been described [57,94], but tend to be significantly less numerous than in FA images, in contrast to other uveitis etiologies such as birdshot chorioretinopathy, Vogt–Koyanagi–Harada disease or sarcoidosis.

### 4.2. Ocular Fluid- and Tissue-Based Diagnostics

Although ophthalmologic examinations may lead to a high suspicion of VRL, differentiation from uveitis remains difficult. Ultimately, diagnostic confirmation through examination of cellular material obtained by invasive procedures is required, unless diagnosis has been obtained by brain biopsy or a positive CSF examination in cases with concomitant CNS involvement. 

Most often, ocular fluids are collected from patients with VRL to make the diagnosis or confirm suspected cancer recurrence. Testing of these fluids may involve cytology, immunophenotyping, protein assays and/or molecular genetic analyses. Typically, the vitreous is sampled for making a diagnosis of VRL [95], but since collection of aqueous humor is simpler and less invasive, it is being used increasingly for any testing with sufficient sensitivity that is required to follow the disease [18,96]. Vitreous (humor) is usually obtained by pars plana vitrectomy, a surgical procedure performed under local or general anesthesia. Surgical instruments are introduced into the posterior segment of the eye, allowing the surgeon to cut and aspirate the viscous vitreous; undiluted or “dry” vitreous is most useful for testing, but any diluted material is also retained for analysis [97,98]. A vitreous “tap,” with a 25- or 27-gauge needle, yields less vitreous and is not commonly used to obtain a specimen for diagnostic purposes. Aqueous (humor) is collected from the anterior chamber by paracentesis, which is readily performed at the slit lamp biomicroscope under topical anesthesia, using a 27- or 30-gauge needle [99]. Retinal biopsy for a confirmation of VRL can usually be avoided.

There continues to be debate around the role of different types of testing of ocular fluids, particularly for the diagnosis of VRL. At many centers, cytology, often combined with flow cytometry, is required to make the diagnosis [100]. However, for both technical and pathological reasons, this can be challenging [101], and the negative predictive value of cytology has been estimated at approximately 60% [102]. Lymphoma cells are scarce and fragile in the vitreous, an issue that is compounded by treatment with corticosteroid drugs, which are often given for suspected uveitis. The viscous nature of the vitreous and the damage to cells during the vitrectomy complicate processing, and a limited sample may need to be shared with the microbiology service while the diagnosis remains in question. Additionally, cytological assessment requires special expertise. The diagnosis of VRL may be delayed for over a year, and multiple procedures are often required to reach the diagnosis [103]. The absolute requirement of cytological confirmation of vitreoretinal lymphoma has therefore been challenged, and it has been suggested that other tools, including cytokine analyses and presence of tumor-specific mutations, might suffice in some situations [104,105]. Combining the results of different pathological investigations [15,106] with multimodal ophthalmic imaging [88] may increase confidence in using alternative molecular tests for diagnosis.

#### 4.2.1. Cytology and Immunophenotyping

Cytological assessment of the vitreous establishes a tissue diagnosis of vitreoretinal lymphoma. Optimal preanalytical conditions and a close collaboration between the surgeon and the pathologist/cytologist are of great importance. The material should either be processed immediately or put in a mild fixative such as HOPE or CytoLyt solution, which preserves cytological detail, immunoreactivity and nucleic acids [107]. Cytological specimens are usually prepared with the cytospin technique. Gonzales and Chan [75] published a useful flow diagram of the pipeline for processing the vitreous for cytology. Cytospins or smears on slides are stained with Papanicolaou, Wright–Giemsa or Diff-Quik stain, and embedding fixed vitreous cells in a paraffin block is an option to increase diagnostic success in cell-rich specimens [108,109]. Well-preserved VRL cases show abundant large atypical lymphoid cells with basophilic cytoplasm and large irregular nuclei with prominent nucleoli. Malignant B cells are considerably larger than benign lymphocytes, with prominent nuclei and scant cytoplasm. In a classical description of vitreoretinal lymphoma, Char et al. [110] described four characteristic cytologic features: “(1) irregular nuclear contours, (2) lobation of nuclei, sometimes giving them a “cloverleaf” appearance (3) coarse irregular chromatin and (4) presence of nucleoli.” However, cases may show large numbers of reactive lymphocytes, cellular debris and macrophages, difficult to discern from a reactive process based on morphology alone. Preceding steroid therapy results in massive degenerative changes and necrosis, but nucleic acids for molecular studies are usually preserved. Of note, atypical cells may occasionally also be observed in reactive conditions, such as viral infections.

The sensitivity of cytology alone reported in the literature ranges from 45% to 81%, with rare false positives [46,111]. In a large study of largely vitreous samples from 100 patients with vitreoretinal lymphoma and 82 patients with uveitis, the United States National Eye Institute investigators reported that cytology had a sensitivity of 81% and a specificity of 99% [112]. However, another large series of 217 patients treated by members of the Japanese Intraocular Lymphoma Study Group [46] indicated positive cytology in just 45% of patients, while a study from Hadassah Hebrew University Medical Center [113] with 150 patients had a sensitivity of 74%. This broad range of sensitivity likely reflects selection bias, but also the lack of the gold standard to define true negatives. Immunophenotyping can be performed either by flow cytometry or immunocytochemistry on cytospins [29,107,114,115]. A predominance of atypical cells expressing B cell markers such as CD20, PAX5 or CD79a and kappa versus lambda light chain restriction by flow cytometry are diagnostic for VRL. Of note, poor cell preservation and a high frequency of reactive cells may limit the diagnostic yield of flow cytometry.

#### 4.2.2. Cytokine Levels

Cytokine testing for diagnosis was first explored over 20 years ago by Chan et al. [116] when interleukin (IL) 10 was measured in vitreoretinal lymphoma and IL-6 was detected in uveitis, in vitreous samples from eight patients. Interleukin 10 is a growth factor for malignant B cells, explaining the high levels in lymphoma [117]. Interleukin 6 is an inflammatory cytokine, which is characteristic of uveitis [118]. As first implemented, the test involved an ELISA of the vitreous fluid: the levels of IL-10 and IL-6 were compared directly, and if greater than one, the result was taken to indicate lymphoma. In one large series of 150 patients with vitreoretinal lymphoma, sensitivity of testing with this interpretation was 92% [46]. Later studies showed that a similar IL-10/IL-6 ratio could be measured in the aqueous, which can be readily collected for serial measurements to follow lymphoma over time [96,119]. Combining aqueous and vitreous testing may improve detection accuracy [113]. The mathematics of this test has been optimized to achieve high detection accuracy. Variations include optimized concentration cutoff values [120], the Hôpital Pitié-Salpêtrière Interleukin Score for Intraocular Lymphoma Diagnosis (ISOLD) [121] and the National Eye Institute Logistic Regression Model [122]. These adjustments resulted in sensitivities of 93–94% and specificities of 95–100% across large cohorts of 119–445 patients. Today, comparison of the two cytokines is routinely used across many countries, both diagnostically and for following patient’s post-diagnosis.

#### 4.2.3. Determination of Clonality by Immunoglobulin Gene Rearrangement Studies

During normal B cell development, rearrangements of the immunoglobulin heavy and light chain genes takes place to ensure that ultimately each B cell will produce a unique antibody [123]. Polymerase chain reaction (PCR) targeting rearranged IG genes yields multiple amplicons if the cell population is polyclonal or inflammatory, and a single amplicon if the cell population is monoclonal, which is the case in malignant lymphoma. First described in the vitreous collected from lymphoma with secondary involvement of the eye by Katai et al. [124], independent groups have confirmed that determination of B cell clonality using consensus primers against immunoglobulin heavy and kappa light chain genes is a valuable adjunct for lymphoma diagnosis and can be performed easily on vitreous aspirates. On average, the sensitivity of standard clonality studies ranges between 46% and 95%, depending on the choice of primer sets [7,15,17,22,112,113]. Since VRL shows a high frequency of somatic hypermutation potentially abrogating primer binding, false negatives can occur even with highly cellular specimens and the optimal technique. On the other hand, due to the immune-privileged location, inflammatory conditions can give rise to oligoclonal or even clonal expansions of nonmalignant lymphoid cells and may lead to false positive results, requiring careful interpretation in the context of other findings [7,15].

It remains to be seen whether NGS-based determination of clonality, which not only separates amplification products by size but relies on unique rearranged sequences, will render superior results.

#### 4.2.4. Mutational Analysis

Recently, detection of recurrent mutations has been shown to increase the diagnostic yield of vitreous specimens. In a study of vitreous samples from 69 patients conducted at University Hospital Tübingen, there was a substantial increase in the sensitivity of the diagnostic pipeline (0.62 to 0.91) for VRL with no impact on specificity; when conducting mutation-specific PCR for *MYD88*, the most frequently mutated gene in PCNSL, it was added to vitreous cytology and *IGH* gene rearrangement studies [15]. Testing for *MYD88* has also been adapted for the aqueous: in a group of 23 patients with vitreoretinal lymphoma and 40 patients with uveitis, vitreous testing had a sensitivity of 75% and specificity of 100%, while aqueous testing had a sensitivity of 67% and specificity of 100% [18]. Serial measurements may be useful to follow the disease [125]. The *MYD88* gene mutation test has been modified as a single-cell zygosity assay [126], and pilot work suggests that single vitreous B cell analyses combining *IGH* gene rearrangement and *MYD88* mutation may have high diagnostic accuracy [127]. Whereas initial studies focused on single or few mutated genes, including *MYD88* and *CD79B* [20,27,100,103,110,128,129], several groups have recently used molecular profiling technologies to identify copy number losses of tumor suppressor genes, including *CDKNA2,* or screen for mutations in multiple candidate genes using next-generation sequencing (NGS) [16,26]. In addition to cellular material, DNA extracted from the cell-free supernatant of vitreous specimens or the aqueous humor are an excellent source of mutation detection either by NGS or digital droplet PCR [18,126]. Of note, cell-free DNA from the vitreous shows high variant allele frequencies, likely due to tumor DNA released from necrotic cells. 

#### 4.2.5. Future Opportunities in Testing

Given the challenges for cytological diagnosis of vitreoretinal lymphoma, there is considerable interest in additional biological disease markers. Over the past 10 years, multiple potential biomarkers have been investigated, including microRNA, B cell regulatory cytokines and survival factors, as well as various immunological proteins. These developments are summarized below, acknowledging that to date, translation into routine clinical practice has not been realized.

In addition to mutation detection, quantitation of microRNAs has shown promise for VRL diagnosis. MicroRNA (miRNA) is a noncoding RNA no longer than 30 base pairs in length that regulates gene expression post-transcriptionally [130]. Different miRNAs have been associated with a broad spectrum of diseases, including cancers. Several independent groups have evaluated miRNA in vitreous samples from patients with vitreoretinal lymphoma. Tuo et al. [131] compared the vitreous from three patients with lymphoma and three patients with uveitis using a 168-miRNA array: mir-155 was significantly lower in lymphoma. Kakkassery et al. [19] assessed expression of three miRNA –miR-19b, miR-21 and miR-92; 0– in the vitreous from 10 patients with lymphoma, as well as from 47 patients with uveitis or epiretinal membrane, and all found distinguished lymphoma, with the highest accuracy for miR-92.

Minezaki et al. [132] studied the vitreous from 14 patients with lymphoma, 40 patients with uveitis, 26 patients with a macular hole or the epiretinal membrane, as well as 12 healthy persons using a 2565-miRNA array. A total of 293 miRNAs were differentially expressed in the vitreous, mostly upregulated, and some were also upregulated in serum: miR-6513-3p, 138-2f-3p and 445-3p were upregulated in both the vitreous and serum, and mir-6513-3p had the greatest discrimination power for lymphoma. Although there are differences in methodology and results across these studies, clearly, miRNA biomarkers are a fruitful area for future research.

Another group of potential biomarkers for VRL are the molecules that control B cell activities. Takeda et al. [133] used protein panels to compare levels of B cell regulatory cytokines in the vitreous from 22 patients with lymphoma and 44 patients with uveitis or epiretinal membrane. As well as IL-10, IL-22 was increased in the vitreous collected from eyes with lymphoma, and IL-35 correlated with worse 5-year survival. The B cell chemokine, CXCL13, is produced by malignant B cells in PCNSL [134] and has been described as a cerebrospinal fluid biomarker [135], suggesting possible value in vitreoretinal lymphoma.

There has recently been interest in computational algorithms that use clusters of proteins detected in ocular fluids to diagnose vitreoretinal lymphoma. Kuiper et al. [136] took aqueous samples from 175 patients, including 27 patients with lymphoma, and used a 27-protein multiplex immunoassay with unsupervised hierarchical clustering to develop a molecular profile that distinguished between lymphoma, retinal detachment, uveitis and macular degeneration on the basis of levels of IL-10, IL-21 and angiotensin-converting enzyme. Nezu et al. [137] collected 512 aqueous samples and studied 28 cytokines by immunoarray, allowing the classification of 17 ophthalmic diseases with a machine-learning algorithm that identified vitreoretinal lymphoma based on concentrations of IL-10, interferon gamma-inducible proteins and angiogenins.

## 5. Treatment

### 5.1. Management of Vitreoretinal Lymphoma

VRL is considered to be a part of central nervous system lymphoma (CNSL). A literature survey on CNS and VRL divided the previous publications into two groups from the ophthalmological point of view, i.e., (141) PCNSL ± ocular involvement and [39] VRL ± non-ocular CNS involvement [138]. In both groups, the managements of VRL should be carried out in collaboration with ophthalmologists and non-ocular experts in the fields of oncology and neurology, such as oncologists, hematologists, neurologists, radiologists and others. Diagnosis, treatment and follow-up of VRL lesions in the eye are taken care of by ophthalmologists, whereas those in the CNS and other systemic organs should be managed by the aforementioned non-ocular experts. Such collaborative medical care is essential for the management and better prognosis of VLR. In 2011, the International PCNSL Collaborative Group published therapeutic principles for VRL [39]. According to the group, no optimal therapy for VRL without detectable CNS lesions has been defined, but systemic therapy is required if lymphoma lesions occur in the CNS and local therapy may be used if the disease is limited to the eye.

This review is focused on local ocular therapy of PVRL and discusses prophylactic systemic therapies to prevent CNS progression.

### 5.2. Local Ocular Therapy for PVRL

According to Wang et al. [139], PVRL patients with one eye involvement can be treated with local ocular therapy which includes external beam radiotherapy and/or intravitreal chemotherapy with methotrexate (MTX) and/or rituximab (anti-CD20 monoclonal antibody). There is no established treatment protocol for PVRL patients with bilateral involvement. However, a previous report recommended systemic therapy with intravenous MTX (8 gm/m^2^ every 2 weeks initially) combined with intravitreal rituximab and MTX [140].

Radiation to the eye is a traditional therapy [141,142] and is still used even today for the therapy of PVRL [43,143,144,145]. In fact, radiation to the eye is highly effective in achieving complete remission of ocular lymphoma with doses ranging from 30 to 45 Gy [139]. However, therapy carries the risk of irreversible radiation retinopathy, optic atrophy and cataract [44,139]. While ocular radiation can be used as an initial local therapy for PVRL, it should not be repeated for recurrent PVRL due to increased risk of irreversible vision loss [44,139]. At present, there are no good controlled studies comparing the two available first-line local therapies (ocular radiation therapy vs. local chemotherapy). Some experts prefer using ocular radiation therapy, and others prefer local chemotherapy [39,44].

Fishburne et al. first reported intravitreal MTX injection as an adjunctive therapy in four patients with VRL [146]. Since then, the efficacy and safety of intravitreal MTX has been supported by many studies [147,148,149,150]. The largest series of VRL patients treated with intravitreal MTX (44 eyes of 26 patients) was reported by Frenkel et al. in 2008 [149]. They injected intravitreal MTX (400 mg/0.1 cc) twice weekly for 4 weeks, once weekly for 8 weeks and then once monthly for 9 months, for a total of 25 injections. According to the study, clinical remission was reached after 6.4 (range, 2–16) MTX injections. Our experience of intravitreal MTX in PVRL patients is the same as their reports. Intravitreal MTX may cause irritation and damage to corneal epithelial cells (corneal epitheliopathy) if intravitreal MTX solution extravasates into the subconjunctival space. One option to decrease this risk is to take 0.1 mL aqueous humor from the anterior chamber for the measurements of intravitreal concentrations of IL-10 and IL-6 prior to the intravitreal MTX injection. This procedure also decreases intraocular pressure, resulting in avoiding extravasation of the MTX solution in the subconjunctival space and preventing corneal epitheliopathy. We adhered to the protocol of Frenkel et al. with modifications: we initiated intravitreal MTX (400 mg/0.1 cc) injections with intervals of twice weekly and measured IL-10 concentration in the anterior chamber at the time of each injection. If VRL lesions disappear or become atrophic, and intraocular IL-10 concentrations decrease below detectable levels, then the interval of intravitreal MTX is extended from twice weekly to once weekly, or from once weekly to once monthly. But more often, MTX is injected without control of IL-10.

More recently, intravitreal rituximab or a combination of MTX and rituximab has been reported to be effective [151,152]. Kitzmann et al. showed 1 mg of intravitreal rituximab in 0.1 mL is safe and effective to treat VRL [151].

### 5.3. Systemic Therapy of PVRL

A major issue in the management of PVRL is whether prophylactic systemic therapy is effective in preventing progression of CNS involvement or in prolonging CNS progression-free survival. A number of retrospective and prospective studies have been carried out to answer this important question and achieve better overall survival. According to a retrospective study by the International PCNS Collaborative Study Group with 83 HIV-negative immunocompetent PVRL patients from 16 centers in seven countries, progression-free survival (from the date of ocular lymphoma diagnosis to the date of the first relapse or progression) and overall survival (from the date of ocular lymphoma diagnosis to death) were 29.6 and 58 months, respectively [143]. There was no statistically significant difference in progression-free survival or overall survival regardless of the treatment modality, and the risk of relapse was similar in the two treatment groups, that is, the local therapy group vs. the extensive therapy group (systemic chemotherapy, whole brain radiation or intrathecal chemotherapy combined with local therapy) [143]. Another retrospective multicenter European collaborative study with 73 patients with PVRL evaluated the outcomes of the following three treatment regimens for PVRL in the prevention of subsequent CNSL [9]: extensive systemic treatment (various combinations of systemic and intrathecal chemotherapy, whole brain radiotherapy and peripheral blood stem cell transplantation) vs. ocular local treatment (ocular radiotherapy and/or ocular chemotherapy) vs. the combination of extensive systemic treatment and ocular local therapy. CNSL developed in 10 of the 31 (32%) patients in the ocular local treatment group, in nine of 21 (43%)—in the extensive systemic treatment group, in nine of 23 (39%)—in the combination treatment group. The 5-year cumulative survival rate was similar among all the treatment groups (*p* = 0.10) [144]. These two retrospective case series studies suggest that extensive systemic treatment does not help to prevent subsequent CNSL. However, at least in the second study, the number of patients for each systemic treatment protocol was rather small to draw conclusions on the long-term effectiveness of these treatment protocols. In addition, all (but only few) patients with stem cell transplantation survived.

On the other hand, there are studies showing significant prophylactic effects of extensive systemic treatments preventing or delaying clinical CNSL involvement [152,153,154,155,156]. A retrospective case series study [153] with 26 patients with PCRL at a single institute evaluated if prophylactic systemic treatment (high-dose systemic MTX and intrathecal MTX together with intravitreal MTX and rituximab) protect against the onset of CNS involvement in PVRL patients. CNS involvement occurred in eight of the 11 patients with prophylactic treatment and in six of the 15 patients without prophylactic treatment, and the difference was not significant. However, the time to onset of CNS lesions in the systemic prophylactic treatment group (42.8 ± 13.8 months) was significantly (*p* = 0.005) longer than that in the group that did not receive prophylactic treatment (10.2 ± 2.0 months), indicating systemic extensive treatment is effective in prolonging CNS progression-free survival time [153]. Another prospective study [154] from a single institute with 10 patients with PVRL treated with intravitreal MTX and systemic high-dose MTX (3.5 g/m^2^, five cycles) and eight PVRL patients treated with intravitreal MTX alone were evaluated for efficacy in preventing CNS involvement. The 2-year CNS lymphoma-free survival was 58% (95% CI, 23.0–82.1%) in the systemic prophylactic treatment group and 37.5% (95% CI, 8.7–67.4%) in the intravitreal MTX alone group, indicating systemic high-dose MTX together with intravitreal MTX seems to be effective in preventing CNS involvement in PVRL [154]. Similar results were seen in a retrospective study from a single center where 59 patients (median age: 70 years) with isolated PVRL were treated with intravenous high-dose MTX as the first-line treatment. However, 8 of the 59 patients also received a local treatment. The brain-free survival was 73 months, showing effectiveness in preventing CNS involvement. Nevertheless, 29 of 59 patients had isolated ocular relapses after the median follow-up of 61 months [155]. Another one-arm prospective study [156] from a single center of 17 patients with PVRL evaluated efficacy of combined treatment with intravitreal MTX, systemic chemotherapy and reduced-dose whole brain radiation therapy in CNS involvement. Four-year progression-free survival was 74.95 and 4-year overall survival was 86.3%, with the median follow-up of 48.9 months, indicating that early prophylactic therapy with systemic chemotherapy and reduced-dose whole brain radiotherapy in PVRL is effective in reducing the CNS relapse rate compared to other previously reported regimens [156].

All in all, due to the rarity of the disease and the various treatment protocols, the available data and publications do not clearly prove the efficacy of prophylactic systemic therapy in preventing CNS involvement in PVRL. An international multicenter study with a large number of patients with PVRL is necessary to establish ideal regimens for the management of PVRL.

## Figures and Tables

**Table 1:** Guideline: consensus recommendations for the diagnosis of vitreoretinal lymphoma [55] (with copyright permission from the publisher).

**(I)** **Clinical history of VRL**
The great majority of patients are 50 years or older. VRL tends to present with floaters and painless loss of vision. It tends not to cause redness or photophobia. VRL is predominantly bilateral but may present initially as unilateral.
**(II)** **Anterior segment findings in VRL**
KPs of various types may be seen in VRL. Anterior synechiae and iris depigmentation are virtually never seen. Scleritis, pseudohypopon, hyphaema, anterior and posterior synechiae are uncommon.
**(III)** **Vitreous and posterior segment findings in VRL**
Vitreous cellular infiltration of any severity is usually seen in VRL. Cells organized into sheets or clumps increase the suspicion of VRL. Snowbanking, vitreous hemorrhage and retinal holes are unlikely signs. VRL patients with retinal lesions tend to show multifocal creamy/white lesions in the outer retina. Other findings include retinal lesions with leopard-skin appearance and RPE atrophy or fibrosis. Retinal hemorrhages, retinal vasculitis, macular edema, retinal detachment and necrotizing retinitis are unlikely, but may be features of advanced disease. Massive vitreous infiltration without macular edema is the most likely presentation for this condition.
**(IV)** **Behavior of VRL with systemic corticosteroids**
Systemic corticosteroids are not appropriate monotherapy for VRL. However typically, VRL improves to some extent on corticosteroids, but may worsen on dose reduction. If clinically feasible, systemic corticosteroids should be discontinued for at least 2 weeks before diagnostic vitrectomy to increase diagnostic yield.
**(V)** **Ocular fluid diagnosis for VRL**
Diagnostic vitrectomy is the preferred investigation for those with clinical suspicion of VRL with vitreous involvement. AC tap for cytokine or gene analysis, where available, may assist diagnosis. Diagnostic vitrectomy with a low cut rate (up to 1500 cuts per minute) is recommended. An early undiluted vitreous sample may also be obtained. Undiluted and diluted vitreous should be immediately analyzed if possible. If not, there should be prior agreement with the pathologist on specimen preservation. Testing vitreous IL-10:IL- 6 is highly recommended. An IL10:IL6 ratio of greater than 1 is a strong indicator of VRL. Testing for MYD88 gene mutation on intraocular fluid is highly recommended. The L265P mutation is a strong indicator of VRL. It is recommended to use cell morphology, immunophenotype (flow cytometry/immunocytology/histochemistry), light chain restriction and IgH and/or TCR gene rearrangements (PCR). If vitreous cytology or flow cytometry are inconclusive after oncologic review by an expert cytopathologist, consider IL10:IL6 or MYD88 analysis, and if not available, consider chorioretinal biopsy or aspirate of suspicious lesions, or repeat vitrectomy. If cytology, flow cytometry, IL10:IL6 and MYD88 mutations are all negative, there is inadequate evidence to support further investigations for B cell lymphoma. If there is strong clinical suspicion of VRL but no supporting cytologic evidence, consider discussion with one of the following: a neurologist, hematologist or oncologist. In situations of diagnostic difficulty, a multidisciplinary approach may be helpful.
**(VI)** **Ocular imaging for VRL diagnosis**
Multi-modal imaging including spectral domain optical coherence tomography (OCT), fundus autofluorescence (FAF), fundus fluorescein angiography (FFA) and indocyanine green (ICG) angiography and B scan ultrasound are recommended when VRL is suspected. Contrast-enhanced MRI of brain is an essential investigation. CT/PET scan of the whole body will exclude systemic involvement. Hypofluorescent round lesions and well-defined hypofluorescent lesions corresponding to the infiltrates in the early and late phases (leopard-spots) on FFA are supportive of VRL. Granular hyper- and hypoautofluorescence pattern is supportive of VRL. Nodular hyperreflective lesions in or beneath the RPE are highly suggestive of VRL on SD-OCT. Widefield fundus photography is a good tool for documentation and follow-up.
